# Antigenized antibodies expressing Vβ8.2 TCR peptides immunize against rat experimental allergic encephalomyelitis

**DOI:** 10.1186/1476-8518-2-9

**Published:** 2004-11-12

**Authors:** Cristina Musselli, Svetlana Daverio-Zanetti, Maurizio Zanetti

**Affiliations:** 1 The Department of Medicine and Cancer Center, University of California, San Diego, La Jolla CA USA

**Keywords:** EAE, TCR, Idiotype, Regulation

## Abstract

**Background:**

Immunity against the T cell receptor (TCR) is considered to play a central role in the regulation of experimental allergic encephalomyelitis (EAE), a model system of autoimmune disease characterized by a restricted usage of TCR genes. Methods of specific vaccination against the TCR of pathogenetic T cells have included attenuated T cells and synthetic peptides from the sequence of the TCR. These approaches have led to the concept that anti-idiotypic immunity against antigenic sites of the TCR, which are a key regulatory element in this disease.

**Methods:**

The present study in the Lewis rat used a conventional idiotypic immunization based on antigenized antibodies expressing selected peptide sequences of the Vβ8.2 TCR (^93^ASSDSSNTE^101 ^and ^39^DMGHGLRLIHYSYDVNSTEKG^59^).

**Results:**

The study demonstrates that vaccination with antigenized antibodies markedly attenuates, and in some instances, prevents clinical EAE induced with the encephalitogenic peptide ^68^GSLPQKSQRSQDENPVVHF^88 ^in complete Freunds' adjuvant (CFA). Antigenized antibodies induced an anti-idiotypic response against the Vβ8.2 TCR, which was detected by ELISA and flowcytometry. No evidence was obtained of a T cell response against the corresponding Vβ8.2 TCR peptides.

**Conclusions:**

The results indicate that antigenized antibodies expressing conformationally-constrained TCR peptides are a simple means to induce humoral anti-idiotypic immunity against the TCR and to vaccinate against EAE. The study also suggests the possibility to target idiotypic determinants of TCR borne on pathogenetic T cells to vaccinate against disease.

## Introduction

Experimental allergic encephalomyelitis (EAE) is an experimentally induced autoimmune disease mediated by T cells. It can be induced in susceptible animals either by immunization with myelin basic protein (MBP) or proteolipid protein PLP, or by immunization with synthetic peptides from the MBP sequence [1]. EAE can also be initiated by the passive transfer of encephalitogenic, MBP-specific T cell lines or clones [2,3]. In the Lewis rat, EAE is characterized by a self limiting, ascending, hind limb paralysis. Histologically, EAE is hallmarked by perivascular and submeningeal infiltration of inflammatory cells within the brain and spinal cord [4]. After recovery, animals become refractory to further induction of paralysis by immunization with MBP. Owing to similarities in clinical expression and histopathology, EAE has long been recognized as an animal model for multiple sclerosis, a demyelinating chronic inflammatory disease in humans of unknown origin. For this reason, studies on EAE are thought to elucidate aspects of the pathogenesis and indicate possible ways of immune intervention.

EAE is mediated by MHC class II -restricted, MBP-specific CD4^+ ^T lymphocytes bearing an antigen receptor (TCR) variable (V) regions belonging to a limited set of TCR V region gene families [5,6] and restricted Vα-Vβ gene combinations [7]. Several rational approaches have been used to prevent EAE, including passive transfer of monoclonal antibodies that interfere with the recognition of the MHC, TCR and MBP peptide complex [8,9], antibodies against CD4 [10] and T regulatory cells [11-14]. Active immunity against attenuated encephalitogenic T cells was shown to prevent the induction of disease [15,16] and vaccination with synthetic peptides of the complementarity-determining regions (CDR) of the TCR of ecephalitogenic T cells, confer resistance to EAE in the rat [17-20]. Together these facts indicated that T cells are crucial to the pathogenesis of EAE and, in converse, immunity to idiotypic determinants of the TCR of encephalitogenic T cells may be protective.

Approaches to directly target the TCR of pathogenetic T cells are an attractive direction for therapy and immunointervention as well as an opportunity to further understand the immunological events involved in protection *in vivo*. However, limitations exist to methods available for TCR vaccination. Vaccination using attenuated encephalitogenic T cells requires that these are specifically expanded *in vitro *and can only be used in an autologous system. Synthetic peptides, albeit successful in several instances [17-20], offer no tri-dimensional conformation and may even yield to opposite effect, *e.g*., worsening of disease [21,22]. Similarly, vaccination with single chain TCR was shown to either prevent or exacerbate EAE in mice [23].

In previous work from this laboratory we demonstrated the induction of anti-receptor immunity using immunoglobulins (Ig) expressing discrete peptide portions of human CD4 [24]. We refer to such Ig as antigenized antibodies, *i.e*., Ig molecules in which foreign peptide sequences are conformationally-constrained and expressed in the complementority-determining region (CDR) loops [25]. Immunization with antigenized antibodies is an efficient method to focus the immune response against defined epitopes of foreign antigens. If CDR sequences of TCRs are functionally comparable to Ig idiotypes, antigenized antibodies provide a tool to induce anti-idiotypic responses against TCR. Here, we used antibodies antigenized with TCR sequences as vaccines to control disease. We engineered two antibodies encompassing in the CDR3 of the heavy (H) chain two synthetic peptides from the sequence of rat Vβ8.2 gene product, ^39^DMGHGLRLIHYSYDVNSTEKG^59 ^(CDR2) and ^93^ASSDSSNTE^101 ^(CDR3, VDJ junction), both reported to confer protection against EAE in the Lewis rat [17-20] when used as vaccines. The results show that vaccination with antigenized antibodies expressing sequences of encephalitogenic T cells induces anti-idiotypic immunity against the TCR and high level resistance against EAE.

## Material and Methods

### Animals

Eight week old, weight-matched female Lewis rats were purchased from Charles River Laboratories (Wilmington, MA). Animals were housed (three rats per cage) in the animal facility of the Universitiy of California, San Diego. Food and water were provided *at libitum*.

### Antigenized antibodies

**Figure 1:**
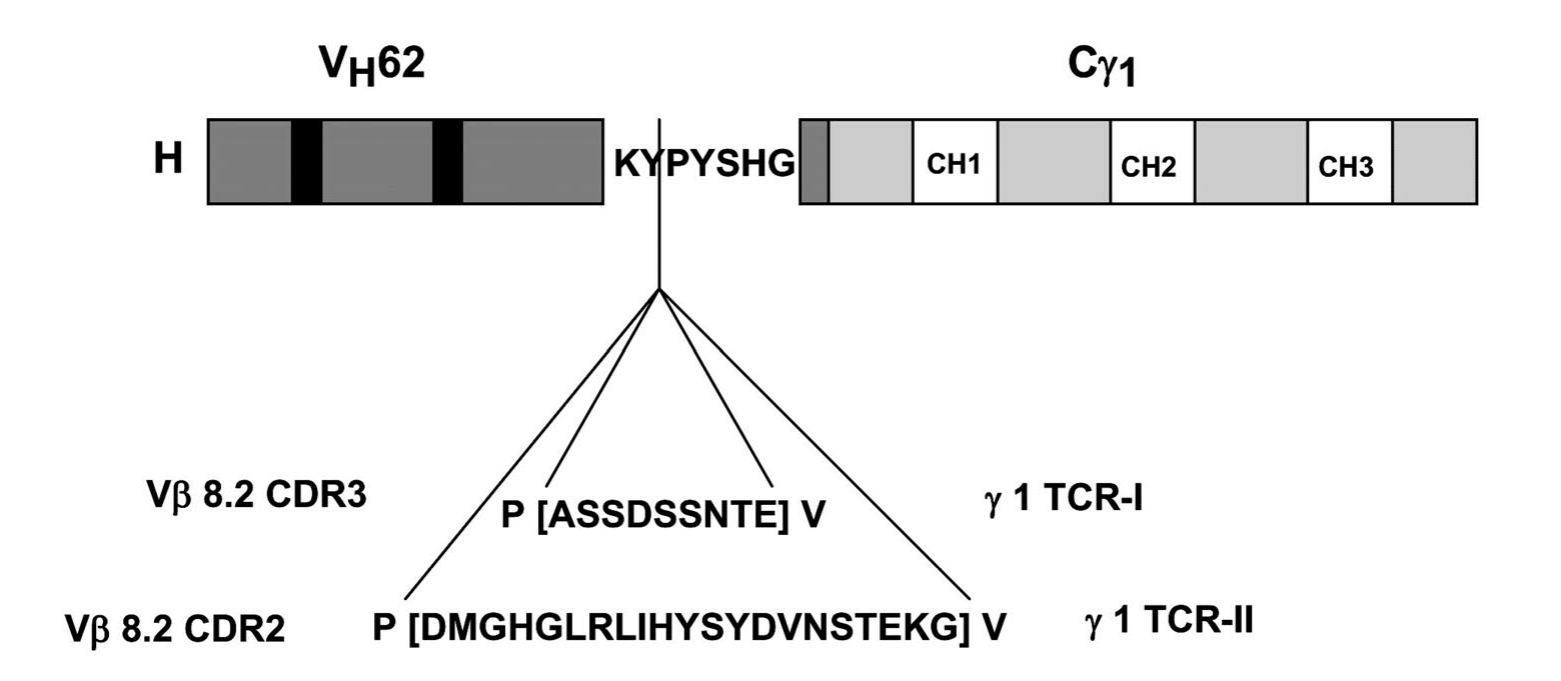
Schematic representation of the two V regions antigenized with TCR sequences. In each case the H chain of the antigenized antibody is formed of a murine V_H_^62 ^region in which the CDR3 has been engineered to express either ^93^ASSDSSNTE^101 ^or ^39^DMGHGLRLIHYSYDVNSTEKG^59 ^sequence between two Val-Pro (VP) doublets of the unique cloning site in the CDR3 loop of V_H_^62^. The complete H chain is the product of the fusion of the antigenized V_H _region with a human γ1C region. The light (L) chain (not shown) is the murine λ1 which is provided by the J558L host cell. (H chain not to scale).

The peptide sequences ^93^ASSDSSNTE^101 ^and ^39^DMGHGLRLIHYSYDVNSTEKG^59 ^were engineered into the CDR3 loop of the murine V_H_^62 ^gene [26] according to our published methods [27]. The antigenized V_H _was then ligated in plasmid vector containing a human γ1 constant (C) region gene. Transfection of the plasmid DNA was performed on murine J558L cells, a H-chain defective variant of myeloma J558, carrying the rearrangement for a λ1 light (L) chain [28]. The resulting antigenized antibodies were termed γ1TCR-I and γ1TCR-II, respectively (Figure 1). Wild-type transfectoma antibodies γ1WT and γ2bWT [26] engineered to have the same C and V regions, but lacking the TCR peptides in the CDR3 of the H chain, served as controls. Transfected cells were incubated without selection for 24 hours and then selected in the presence of 1.2 mg/ml G418 (GIBCO). G418-resistant clones secreting high level of Ig were identified by enzyme-linked immunosorbent assay (ELISA) using horseradish peroxidase (HRP)-conjugated goat antibody to human Ig (Sigma) [29]. Cultures secreting 10–20 μg/ml were selected, expanded, and their supernatants precipitated by (NH_4_)_2_SO_4_. Antibodies were purified by affinity chromatography on a Protein A-Sepharose column (Pharmacia-LKB, Alameda, CA) equilibrated with 3 M NaCl/1M glycine, pH 8.9. Elution was performed using glycine 0.1 M- HCl/0.5 M NaCl pH 2.8. The eluted fractions were neutralized using 1 M Tris-HCl, pH 8.0, and dialyzed against 0.15 M phosphate-buffered saline (PBS) pH 7.3. The purity of the antibodies was assessed by electrophoresis on a 10% Sodium Dodecyl Sulfate (SDS)-Polyacrylamide Gel (PAGE).

### Synthetic peptides

Synthetic peptide GSLPQKSQRSQDENPVVHF corresponding to amino acid residues 68–88 of guinea-pig MBP [30], DMGHGLRLIHYSYDVNSTEKG corresponding to amino acid residues 39–59 of rat Vβ8.2 (CDR2 peptide), ASSDSSNTE corresponding to amino acid residues 93–101 of rat Vβ8.2 (CDR3 peptide) rat [17,18], and the (NANP)_3 _peptide of *Plasmodium falciparum *parasite [31] were all synthesized in the Peptide Synthesis Facility of the Universitiy of California, San Diego. After synthesis peptides were analyzed by HPLC for purity. Peptide KKSIQFHWKNSNQIKILGNQGSFLTKGPS corresponding to residues 21–49 of the extracellular domain of human CD4 was described previously [32].

### Enzyme-linked immunosorbent assay (ELISA)

Serum antibodies against antigenized antibodies and their control were determined by ELISA on 96-well polystyrene microtiter plates (Costar, Cambridge, MA) coated (5 μg/ml – 50 μl/well) with γ1TCR-I, γ1TCR-II, γ2bTCR-I proteins in 0.9% NaCl by drying at 37°C. The wells were blocked with a 1% bovine serum albumin (BSA) in phosphate-buffered saline (PBS), and then incubated overnight at +4°C with individual rat sera diluted in PBS containing 1% BSA and 0.05% Tween 20 (PBSA). After washing, the bound antibodies were detected by adding peroxidase-conjugated goat antibodies to rat IgG (γ specific) (Biomeda, CA) at 1:500 dilution in PBSA for 1 hour at room temperature. After washing, the bound peroxidase was measured by adding o-phenylenendiamine (100 μl/well) and H_2_O_2_. After 30 minutes, the plates were read in a micro-plate reader (Vmax, Molecular Devices) at 492 nm. Tests were done in duplicate. Antibodies to TCR peptides were detected in ELISA on 96-well polystyrene microtiter plates coated (10 μg/ml) with the Vβ8.2 synthetic peptides ^39^DMGHGLRLIHYSYDVNSTEKG^59 ^and ^93^ASSDSSNTE^101 ^in 0.1M carbonate buffer, pH 9.6, by overnight incubation at +4 C. After blocking unreactive sites, sera (1:25 dilution in PBSA) were added to plates and incubated overnight at +4°C. The bound antibodies and reactive peroxidase were detected as detailed above. Ig reactive with synthetic peptide ^21^KKSIQFHWKNSNQIKILGNQGSFLTKGPS^49 ^of human CD4 were determined on 96-well polystyrene microtiter plates coated (2.5 μg/ml) with peptide 21–49 in 0.9% NaCl by drying at 37 C as previously established [32]. Briefly, sera (1:400 dilution in PBSA) were incubated overnight at +4°C. After washing, the test was continued as specified above. Plates were read in a micro-plate reader (Vmax, Molecular Devices) at 492 nm.

### FACS analysis

Autoantibodies reactive with the Vβ8.2^+ ^TCR were sought by flowcytometry on the S23B1E11 T cell hybridoma [33], derived from the fusion of Vβ8.2^+ ^CD4 T lymphoblasts specific for MBP with the murine TCR α/β^- ^BW1100.129.237 thymoma cell line [33]. For FACS analysis the following procedure was utilized. 10^6 ^hybridoma T cells in 100 μl of RPMI-1640 containing 1% egg albumin, 0.01% NaN_3 _and 10 mM Hepes, were incubated with rat sera (1:10 dilution) for 90 minutes at +4°C. Cells were washed three times with cold RPMI-1640 and subsequently incubated with a fluorescein-isotyocianate (FITC)-conjugated goat antibody (0.5 μg/10^6 ^cells) to rat Ig (H+L) (Caltag, So. San Francisco, CA) for 20 minutes at +4°C. After incubation, the cells were washed twice, resuspended in 1% paraformaldehyde, and analyzed in a FACS Scan (BD Biosciences). To stain for dead cells, 20 μl of propidium iodide in PBS were added to unfixed cells before FACS analysis. R-phycoerythrin conjugated mouse monoclonal antibody R78 (IgG1, k) specific for the rat Vβ8.2, the kind gift of Pharmingen (San Diego, CA), was used to control for the expression of the Vβ8.2 TCR on S23B1E11 hybridoma cells.

### *In vitro *proliferative response

Poplyteal, inguinal and paraortic lymph nodes were removed from immunized animals at different times, dissociated and washed in RPMI-1640. Lymph node cells were plated in round-bottom 96-well plates at 2.5 × 10^5 ^cells/well in the presence of various (10–100 956;g/ml) amounts of antigen in 200 μl of RPMI containing 10% FCS, 100 U/ml penicillin, 100 μg/ml streptomycin, 4 mM glutamine, 0.1 mM non-essential aminoacids, 1 mM sodium pyruvate and 0.5 μM 2-β mercaptoethanol. Cultures were incubated for 72 hours in a 10% CO_2 _atmosphere. The evening before harvest 1 μCi/well of [^3^H]-thymidine was added to each well. Cells were harvested onto glass fiber filters and counted on an automatic Beckman LS 6000IC β-counter.

### Vaccinations and immunizations schedule

Animals were vaccinated with antigenized antibodies (100 μg/rat) in complete Freunds' adjuvant (CFA) divided equally between the posterior paws (25 μl each) and two points in the back subcutaneously. A booster injection (50 μg/rat) in incomplete Freunds' adjuvant (IFA) was given subcutaneously on day 21. EAE was induced on day 28 by immunization with MBP peptide ^68^GSLPQKSQRSQDENPVVHF^88 ^(30 μg/rat) in the anterior paws (25 μl each) in CFA (H37RA 10 mg/ml). Control rats were similarly injected with transfectoma antibody γ1WT or γ2bWT. Rats inoculated with Freunds' adjuvant only served as additional control. Serum samples were collected from the retro-orbital sinus on day 0 before vaccination, day 21 before booster injection, day 28 before EAE induction, and day 50 after recovery from disease. Sera were stored at -20 C until use.

### Clinical evaluation of EAE

EAE was monitored daily by two operators for clinical signs using the following scale: grade 0 = no appreciable symptoms; grade 1 = tail atony; grade 2 = paraparesis; grade 3 = paraplegia; grade 4 = paraplegia with forelimb weakness, moribund state. Typically symptoms of disease began to appear on day 11–13 from the injection of the encephalitogenic peptide. The Disease Index was calculated according to the formula: [(Maximum Score) × (Duration of Disease) × (Incidence)].

### Statistical Methods

Statistical analyses was performed using the Fisher's test.

## Results

### Vaccination with antigenized antibodies and effect on EAE

Two antigenized antibodies were engineered to express the CDR3 ^93^ASSDSSNTE^101 ^and CDR2 ^39^DMGHGLRLIHYSYDVNSTEKG^59 ^sequences, and were termed γ1TCR-I and γ1TCR-II, respectively (Figure 1). Rats were vaccinated with an individual antigenized antibody and received a booster injection 21 days later. EAE was induced on day 28 by immunization with the encephalitogenic MBP peptide ^68^GSLPQKSQRSQDENPVVHF^88^. As shown in Table 1, vaccination with both γ1TCR-I and γ1TCR-II reduced disease severity. Rats immunized with γ1TCR-I (group I) had a disease index of 1.8. Within this group, two out of six rats (33%) did not develop disease, one had grade 1 and three had grade 2. None proceeded through grade 3 or 4. Rats immunized with γ1TCR-II (group II) had a disease index of 4.9. Within this group two out of ten rats (20%) did not develop the disease, two had grade 1, four had grade 2 and two had grade 3. In contrast, all fifteen control rats vaccinated with γ1WT or given CFA only (groups III and IV) developed EAE with a disease index ranging between 11.3 and 22.4. Unmanipulated rats immunized with the MBP peptide (group V) developed EAE with a disease index of 25.2. There was a direct correlation between the severity of the disease and its duration. In rats immunized with γ1TCR-I, the disease lasted on average for 2.5 days and in rats immunized with γ1TCR-II 3.8 days. In contrast, in all the other groups (groups III-V) the duration of the disease was significantly longer (6–7 days). Of note, although group III rats had an overall lower score than unmanipulated rats, they differed from rats in group I or group II by the above mentioned parameters and these difference were statistically significant (Table 1). CFA did not confer protection. Taken together, these data indicate that active immunity elicited with antigenized antibodies expressing rat Vβ8.2 TCR peptides was effective in markedly reducing the severity of EAE in the Lewis rat.

**Table 1:** Vaccination against antigenized antibodies expressing TCR peptides protects from EAE

			Severity of Disease*			
			
**Group**	**No. Rats**	**Immunogen**	**Incidence**	**Max Score (mean ± SD)**	**Duration (mean ± SD)**	**Disease Index**
I	6	γ1TCR-I	4/6	1.1 ± 0.9^a^	2.5 ± 2.2^b^	1.8
II	10	γ1TCR-II	8/10	1.6 ± 1.0^c^	3.8 ± 2.3^d^	4.9
III	10	γ1WT	10/10	2.2 ± 0.9	5.1 ± 1.0	11.3
IV	5	CFA	5/5	3.4 ± 0.9	6.6 ± 1.3	22.4
V	6	-	6/6	3.5 ± 0.5	7.2 ± 1.3	25.2

* EAE was scored according to incidence, severity and duration. Disease index was calculated as follows: Mean Maximum Score × Mean Duration Disease × Incidence.

Significance: (^a^) Group I vs Group III p = 0.04 and Group I vs Group V p = 0.0002; (^b^) Group I vs Group III p = 0.009 and Group I vs Group V p = 0.0001; (^c^) Group II vs Group III p = 0.16 and Group II vs Group V p = 0.0005; (^d^) Group II vs Group III p = 0.12 and Group II vs Group V p = 0.001.

### Antibody responses after vaccination

**Figure 2:**
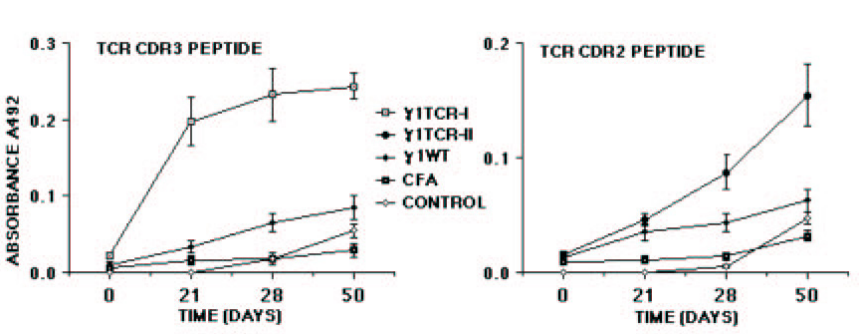
Antibody response to TCR peptides following vaccination with antigenized antibody γ1TCR-I or γ1TCR-II ^39^DMGHGLRLIHYSYDVNSTEKG^59 ^tested on the ASSDSSNTE (panel a) or (panel b). The number of rats in each group is that indicated in Table 1. Results are expressed as Log2 ± SD.

**Figure 3:**
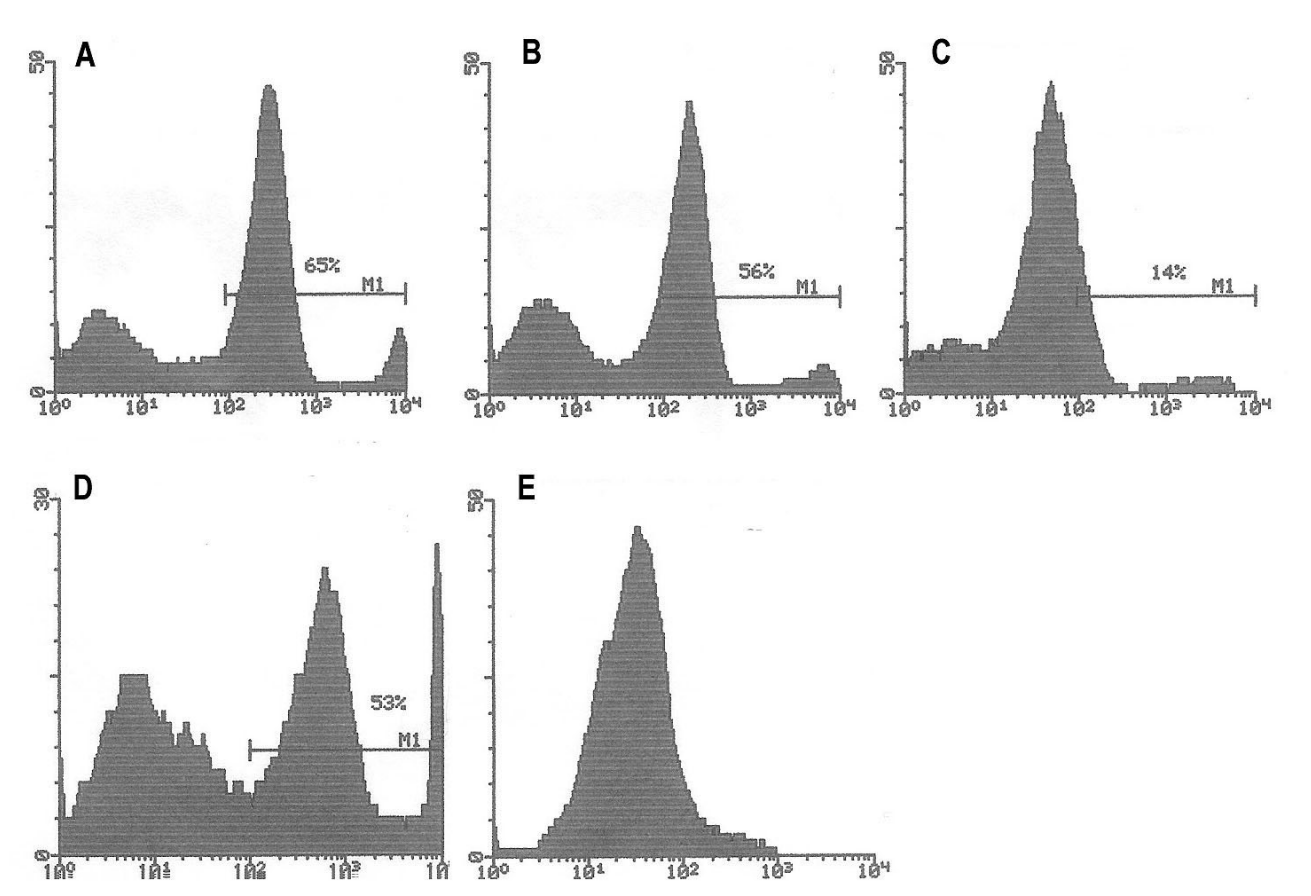
Sera from rats vaccinated with γ1TCR-I bind Vβ8.2^+ ^T cells by flowcytometry. Vβ8.2^+ ^S23B1E11 T cell hybridoma were used as substrate. Sera were tested at 1:25 dilution. Bound antibodies were revealed using a FITC-conjugated goat antibody to rat Ig.

Antibodies in response to the immunogen were assessed by solid-phase ELISA at various times after immunization. As shown in Table 2, antibody titers against the immunogen developed in each group (group I-III) irrespective of which antibody was used to detect the antibody response in sera. This suggests that the human constant region of the antigenized antibodies is immunogenic in the rat. Antibody titers increased after the booster immunization and after challenge with the encephalitogenic MBP peptide. Control rats (group IV-V) did not mount any antibody response. No reactivity was found on the 19^mer ^MBP peptide (GSLPQKSQRSQDENPVVHF) used as a control. Anti-TCR (anti-idiotypic) antibodies were tested using two approaches. In the first case, sera of immunized animals were tested on Vβ8.2 synthetic peptides by ELISA. A weak but distinct response was detected in both instances starting on day 21 or 28 (Figure 2). Sera from control animals did not react with TCR peptides. Together with the fact that these were tested at a 1:25 dilution it appears that the anti-idiotypic response is weak. In the second case, we tested anti-idiotypic antibodies for their reactivity with the TCR in its native configuration. This was done by flowcytometry using the Vβ8.2^+ ^T cell hybridoma S23B1E11 as the cell substrate. Two out of six rats in group I had a bright cellular staining (Figure 3). Reactive antibodies were detectable on day 21, 28 and day 50. Rats immunized with γ1TCR-II (group II) as well control rats (group III-V) were negative. Interestingly, the two rats whose sera reacted with TCR by flowcytometry did not develop symptoms of EAE.

**Table 2:** Detection of antibodies against γ1TCR-I and γ1TCR-II in vaccinated Lewis rats

				**Days After Vaccination**			
				
**a**	**Immunogen**	**Rats (No.)**	**Responders (No.)**	**0**	**21**	**28**	**50**
	
	γ1TCR-I	6	6/6	≤2.3*	3.9 ± .2	4.2 ± .2	4.5 ± 0.2
	γ1TCR-II	10	10/10	≤2.3	3.7 ± 0.2	4.1 ± 0.1	4.5 ± 0.2
	γ1WT	10	10/10	≤2.3	3.2 ± 0.4	3.9 ± 0.2	4 ± 0.2
	CFA	5	0/5	≤2.3	≤2.3	2.6	≤2.3
	-	6	0/6	-	-	≤2.3	≤2.3
**b**							
	γ1TCR-I	6	6/6	≤2.3	4 ± 0.2	4 ± 0.2	4.6 ± 0.3
	γ1TCR-II	10	10/10	≤2.3	4.1 ± 0.3	4.4 ± 0.3	4.7 ± 0.5
	γ1WT	10	10/10	≤2.3	3.3 ± 0.3	4 ± 0.2	4.2 ± 0.2
	CFA	5	0/5	≤2.3	≤2.3	≤2.3	≤2.3
	-	6	0/6	-	-	≤2.3	≤2.3

* Antibody titers are expressed in log10. Sera were tested on microtiter plates coated with each of the TCR antigenized antibody γ1TCR-I (panel a) or γ1TCR-II (pane b). End point dilutions were determined as the last serum dilution binding with an OD ≥ 0.200.

### Vaccination with a murine antigenized antibody

To explore the importance of foreigness of the constant region on the immunogenicity of the Vβ8.2 peptides we engineered an antigenized antibody with a murine γ2b constant region. Homology search using the BLAST program of the NCBI gene bank indicated that the murine γ2b C region is 56.7% identical to the rat γ2b C region, with a homology of 71% between residues 106 and 333. Because significant protection was found in rats vaccinated with the antibody expressing the ^93^ASSDSSNTE^101 ^peptide (γ1TCR-I), we engineered an antibody with the same V region (γ2bTCR-I). Rats vaccinated with γ2bTCR-I and subsequently immunized with MBP peptide, were protected only partially compared to rats vaccinated with γ1TCR-I (10.2 vs. 1.9) (Table 3). Notably, within the six rats immunized with γ2bTCR-I, two were grade ≤ 2 and four developed a grade 3 for an average of two days. On the other hand, three out of six rats immunized with control antibody γ2bWT proceeded through a grade 4 disease. Similarly, all five control rats (group III and IV) developed a grade 4 disease. Of note, although the severity of the disease in group I rats was less than in control group II, the difference was not statistically significant (Table 3). All rats developed antibodies to the respective immunogen. However, when compared with the total antibody titer of rats immunized with γ1TCR-I and γ1TCR-II the titers were on average lower at single time points (Table 4). All sera reacted with the synthetic peptide ^93^ASSDSSNTE^101 ^starting from day 21 with a progressive increase over time (Figure 3).

**Table 3:** Protection against EAE by vaccination with antigenized antibodies with a murine γ2b constant region

			Severity of Disease*			
			
**Group**	**No. Rats**	**Immunogen**	**Incidence**	**Max Score (mean ± SD)**	**Duration (mean ± SD)**	**Disease Index**
I	6	**γ2bTCR-I**	6/6	2.5 ± 0.8^a^	4.2 ± 0.4^b^	10.5
II	6	**γ2bWT**	6/6	3.0 ± 1.3	5.7 ± 1.7	17.1
III	2	**CFA**	2/2	4	6	24
IV	3	-	3/3	4	7.3 ± 0.6	29.2

* EAE was scored according to incidence, severity and duration. Disease index was calculated as follows: Mean Maximum Score × Mean Duration of Disease × Incidence.

*Significance: (^a^) Group I vs Group II p = 0.394 and Group I vs Group IV p = 0.051; (^b^) Group I vs Group III p = 0.05 and Group I vs Group IV p = 0.026*.

**Table 4:** Detection of antibodies against γ2bTCR-I in vaccinated Lewis rats

			**Days After Vaccination**			
			
**Immunogen**	**Rats (No.)**	**Responders (No.)**	**0**	**21**	**28**	**50**
**γ2bTCR-I**	6	6/6	≤2.3	3.2 ± 0,2	3.7 ± 0.1	4 ± 0.3
**γ2bWT**	6	6/6	≤2.3	3 ± 0.3	3.4 ± 0.3	3.6 ± 0.3
**CFA**	2	0/2	≤2.3	≤2.3	≤2.3	≤2.3
**-**	3	0/3	-	-	≤2.3	≤2.3

* Antibody titers are expressed in log10. Sera were tested on microtiter plates coated with γ2bTCR-I. End point dilutions were determined as the last serum dilution binding with an OD ≥ 0.200.

### Serum antibodies of vaccinated rats bind a synthetic peptide of human CD4

**Figure 5:**
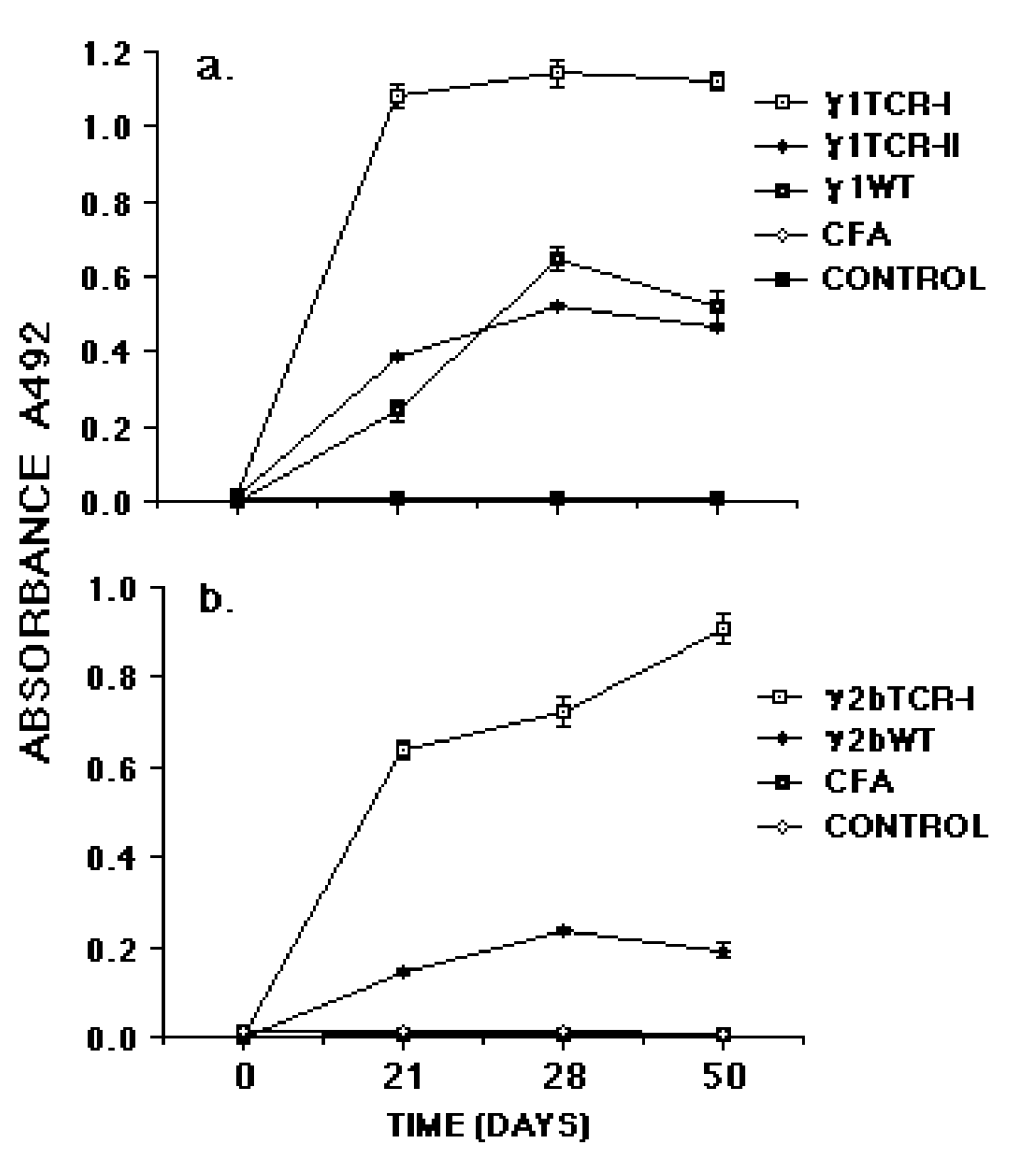
Sera from rats vaccinated with γ1TCR-I or γ2bTCR-I bind synthetic peptide 21–49 of human CD4.

In the attempt to correlate the antibody response after vaccination with protection, the sera of vaccinated rats and their controls were tested on a synthetic peptide corresponding to amino acid residues 21–49 of the first extra-cellular domain of human CD4. This peptide binds Ig irrespective of antigen specificity and heavy chain isotype with an affinity of 10^-5 ^M (26). It also binds antigen:antibody complexes formed at molar equivalence with an affinity about 100 fold higher [31]. When the sera of vaccinated rats were assayed on plates coated with the synthetic peptide of human CD4, strong binding was observed by sera from all rats immunized with γ1TCR-I whereas sera from rats immunized with γ1TCR-II or γ1WT bound much less (Figure 5a). Control sera of groups IV and V did not bind. Binding could be attributed either to a differential property of the two antigenized V regions or to differences in the immune response triggered by the V regions themselves. To distinguish between the two possibilities two experiments were performed. First, we assessed binding of γ1TCR-I and γ1TCR-II on the CD4 peptide. Both bound equally at saturating and non-saturating concentrations (data not shown). Second, we tested sera of rats immunized with γ2bTCR-I considering that, if the effect was due to the immune response against ^93^ASSDSSNTE^101^, we would have found similar results. As shown (Figure 5b), the sera of γ2bTCR-I vaccinated rats all bound to the CD4 peptide comparably to rats vaccinated with γ1TCR-I. This suggests that binding to the CD4 peptide may reflect differences in the type of V regions utilized by the antibodies generated *in vivo *in response to immunization with the TCR peptide ^93^ASSDSSNTE^101 ^as compared with the TCR peptide ^39^DMGHGLRLIHYSYDVNSTEKG^59 ^or the wild type V region. Further studies will be needed to clarify this issue.

### Proliferative response

Spleen cells and draining lymph nodes of rats tested 15 or 30 days after the initial immunization were tested in a proliferative assay against the Vβ8.2 peptides. No proliferative response was detected (data not shown).

## Discussion

In this report we demonstrate that the severity of EAE in the Lewis rat can be greatly attenuated, and in some instances completely prevented, by active immunization with antigenized antibodies expressing amino acid sequences of the rat Vβ8.2 gene product. Immunity against synthetic peptides of the TCR has been shown to be effective in preventing or reducing the severity of EAE in the rat [17-20], suggesting that autoimmunity against the TCR reacting with encephalitogenic sequences of MBP is key to immunoregulatory events. The control of pathogenetic T cells may involve both T cells and antibodies. Autoregulation via T cells in EAE is well established. Thus, spontaneous recovery from EAE is impaired by splenectomy or thymectomy [34] and EAE can be prevented by vaccination with "attenuated" pathogenic T cells [15]. Autoregulation in EAE may involve both CD4^+ ^and CD8^+ ^T cells [35-38] as well as suppression by cytolytic T-T interactions [39]. A prevailing idea has been that in the rat [40] and in the mouse [41] idiotypic determinants of the TCR may be autoimmunogenic and contribute to mechanisms of immune regulation leading to protection. On the other hand, at least in a few instances, monoclonal antibodies against these TcR Vβ region [9,42,43] or against TCR idiotype [44] have been shown to block or attenuate disease.

Here we show that immunity against idiotopes of antibodies engineered to express TCR peptides is effective in generating anti-idiotypic immunity directed against rat Vβ8.2 TCR gene product. Importantly, this type of immunity protected from EAE. The new approach used herein to induce anti-TCR immunity is based on conventional idiotypic immunization in which antigenized antibodies mimic the immunogenic properties of soluble TCR functioning as a surrogate internal image [45] in much the same way as previously demonstrated for a non-self antigen [31]. The present approach is reminiscent of experiments in which induction of anti-idiotypic immunity against TCR with specificity for MHC was obtained by immunization with soluble alloantibodies of relevant specificity [46,47], or by immunization with autologous idiotype positive molecules that are shed from the cell surface in the serum [48]. Thus, antibodies purposely modified to express selected loops of the TCR obviate the necessity to purify the receptor, isolate idiotypic TCR molecules from the serum, or use antigen-specific T cell blasts.

Antibodies reacting with TCR peptides were detected in every vaccinated rat indicating that immunization with antigenized antibodies is an efficient method to induce an anti-idiotypic response specific for a somatic receptor. The fact that only two out of sixteen vaccinated rats developed antibodies against the native receptor detectable by flowcytometry on Vβ8.2^+ ^T cells suggests that cross-reactive anti-idiotypic antibodies may be very low titer. Alternatively, they may be adsorbed on T cells *in vivo *precluding their detection in the serum. The first possibility is consistent with the self nature of TCR peptides and a predicted paucity of self reactive clonotypes within the natural B cell repertoire. Interestingly, we noted that the anti-idiotypic response against a non-self peptide expressed in an antigenized antibody [31] is much greater than the one observed here against a self peptide. That only rats vaccinated with the antigenized antibody expressing the ^93^ASSDSSNTE^101 ^sequence developed flowcytometry-reactive autoantibodies could reflect difference in conformation once the two peptides are embedded in the CDR3 loop of an antigenized antibody. For instance, ^93^ASSDSSNTE^101 ^could be better surface exposed and more stably expressed ^39^DMGHGLRLIHYSYDVNSTEKG^59^. A computer-assisted comparison of hydrophilicity profiles [49] of the ^93^ASSDSSNTE^101 ^peptide in the parental. TCR Vβ8.2 gene product and in the antibody V region shows that in both instances the peptide is highly hydrophilic (Figure 5). On the other hand, the Vβ8.2 CDR2 region shows a highly hydrophilic profile alternating with large hydrophobic regions of poorly exposed amino acid residues, both in the parental TCR and in the antibody CDR (data not shown).

Our data show that although the process of antibody antigenization allows one to conformationally-constrain and express discrete peptide sequences of somatic receptors, the induction of anti-receptor antibodies is not directly predictable. Previously, we demonstrated flowcytometry-reactive antibodies to human CD4 in a high proportion (75 %) of cases [24]. We conclude that the physical characteristics of a given receptor peptide (e.g., length, hydrophilicity, etc.) likely determine its ability to induce antibodies cross-reactive with the native receptor.

Interestingly, rats immunized with the antigenized antibody expressing ^93^ASSDSSNTE^101 ^but not ^39^DMGHGLRLIHYSYDVNSTEKG^59 ^reacted immunologically with a synthetic peptide of human CD4 previously described to bind Ig [32]. Because the two antigenized antibodies reacted equally with the CD4 peptide and only differ by the composition of their CDR3, we suggest that binding to CD4 by anti- ^93^ASSDSSNTE^101 ^serum antibodies underscores qualitative differences of the immune response between rats immunized with γ1TCR-I and 1TCR-II, respectively. Thus, it appears as if ^93^ASSDSSNTE^101 ^induced a different immune response than ^39^DMGHGLRLIHYSYDVNSTEKG^59^. Furthermore, since vaccination with γ1TCR-I also promoted greater protection from EAE, it is tempting to speculate that a component of the anti-idiotypic response against γ1TCR-I is associated with protection.

In conclusion, three points have emerged from this study. First, antigenized antibodies expressing conformationally-constrained loops of the Vβ8.2 TCR can be used as vaccines in the prevention of EAE in the Lewis rat. Our new approach to generate anti-TCR immunity, confirms the relevance of anti-idiotypic regulation in controlling rat EAE [17,18,20]. Second, since a weak antibody anti-idiotypic response in the apparent lack of a cell proliferative response was associated with protection, it appears as if a humoral anti-TCR response is relevant to protection from disease. Although this contrasts the relevance of T cell immunity in the regulation of EAE in the rat, reports exist to support the idea that humoral immunity is also important [20,50,51]. EAE was shown to be prevented or attenuated by passive transfer of serum from rats recovering from EAE [52], or by passive transfer of monoclonal antibodies against these TCR Vβ region and its idiotypes [9,42-44]. However, whether anti-idiotypic antibodies against the TCR predispose to anergy, apoptosis or killing of pathogenetic T cells remains to be determined. Finally, our study indicates that antigenized antibodies can be used as vaccines in conditions where immunopathology and disease involve receptors on somatic cells, and anti-receptor immunity alone could prevent or mitigate a pathological condition.

## Competing interests

The authors declare that they have no competing interests

**Figure 4:**
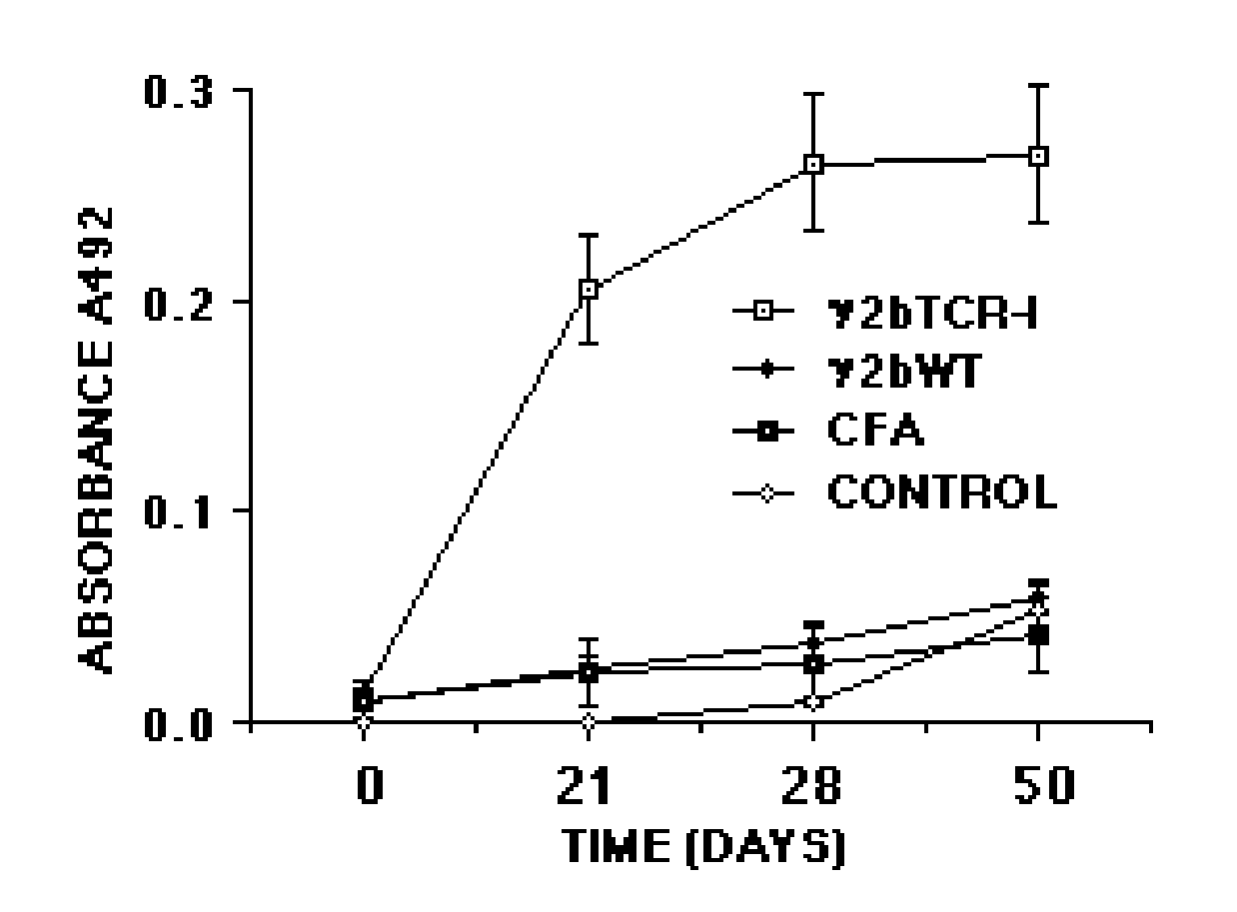
Antibody response to TCR peptide ^93^ASSDSSNTE^101 ^following vaccination with antigenized antibody γ2bTCR-I. The number of rats in each group are not indicated in Table 3. Results are expressed as means of Log2 ± SD.

**Figure 6:**
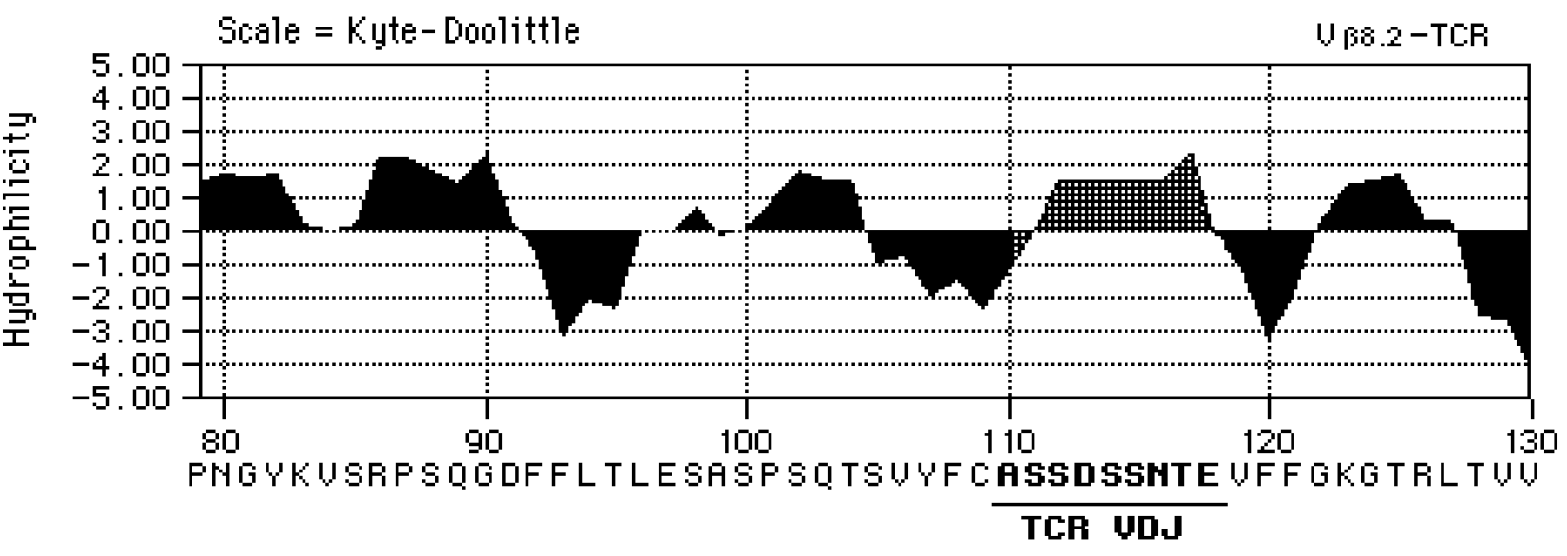
Hydrophilicity profiles of TCR peptides-containing V regions. Hydrophilic profile of the rat Vβ8.2 TCR, amino acid residues 80–130, inclusive of the CDR3 sequence ^93^ASSDSSNTE^101^.

**Figure 7:**
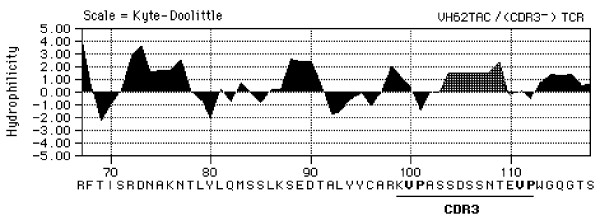
Hydrophilic profile of the mouse VH62, amino acid residues 80–125, engineered with the ^93^ASSDSSNTE^101 ^peptide of the rat Vβ8.2 TCR-CDR3.
